# A Gap Junction Circuit Enhances Processing of Coincident Mechanosensory Inputs

**DOI:** 10.1016/j.cub.2013.04.030

**Published:** 2013-06-03

**Authors:** Ithai Rabinowitch, Marios Chatzigeorgiou, William R. Schafer

**Affiliations:** 1 Cell Biology Division, MRC Laboratory of Molecular Biology, Francis Crick Avenue, Cambridge CB2 0QH, UK

## Abstract

Electrical synapses have been shown to be important for enabling and detecting neuronal synchrony in both vertebrates [1–4] and invertebrates [5, 6]. Hub-and-spoke circuits, in which a central hub neuron is electrically coupled to several input neurons, are an overrepresented motif in the *C. elegans* nervous system [7] and may represent a conserved functional unit. The functional relevance of this configuration has been demonstrated for circuits mediating aggregation behavior [8] and nose touch perception [9]. Modeling approaches have been useful for understanding structurally and dynamically more complex electrical circuits [10, 11]. Therefore, we formulated a simple analytical model with minimal assumptions to obtain insight into the properties of the hub-and-spoke microcircuit motif. A key prediction of the model is that an active input neuron should facilitate activity throughout the network, whereas an inactive input should suppress network activity through shunting; this prediction was supported by cell ablation and in vivo neuroimaging experiments in the *C. elegans* nose touch circuit. Thus, the hub-and-spoke architecture may implement an analog coincidence detector enabling distinct responses to distributed and localized patterns of sensory input.

## Highlights

• A model hub-and-spoke circuit defines a role for shunting in sensory processing
• Nonrectifying gap junctions allow inactive neurons to inhibit network activity
• Shunting and lateral facilitation both contribute to nose touch perception
• The hub-and-spoke microcircuit mediates analog coincidence detection

## Results

**Figure 1:**
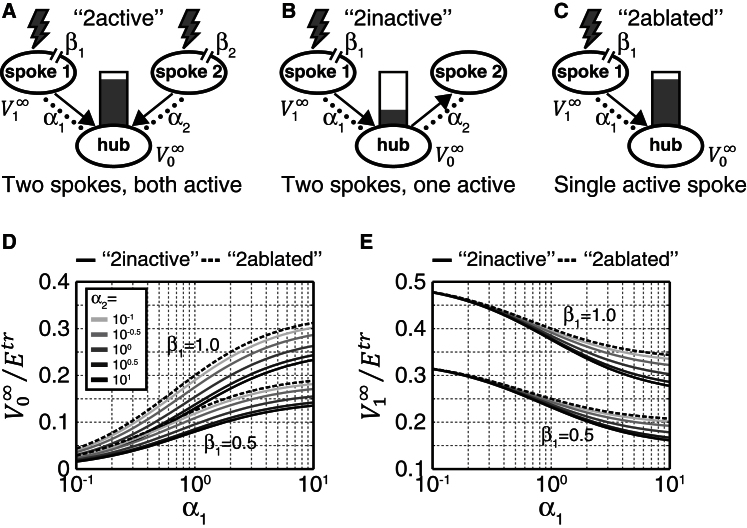
Hub-and-Spoke Circuit Model (A) Model of a hub-and-spoke circuit (see Supplemental Experimental Procedures). β_1_ and β_2_ are the relative transduction strengths of spokes 1 and 2 in the presence of sensory stimuli (lightning symbols). α_1_ and α_2_ are the relative coupling strengths of the gap junctions connecting spokes 1 and 2 to the hub (dotted lines). $V_{1}^{\infty }$ and $V_{0}^{\infty }$ are the steady-state membrane potentials of spoke 1 and the hub, respectively. Arrows indicate net direction of current flow, and the magnitude of $V_{0}^{\infty }$ is represented schematically by the size of the gray bar. (B) When just one input is received in spoke 1 (lightning symbol), entailing an inactive spoke 2 (“2inactive”) implemented in the model by setting β_2_ = 0, $V_{0}^{\infty }$ is expected to decrease in size, as illustrated by the shortened gray bar, since current now flows in the opposite direction from the hub to spoke 2 (arrows indicate net direction of current flow). (C) If an input is received in spoke 1 (lightning symbol) but spoke 2 is removed from the circuit altogether (“2ablated”), implemented by setting α_2_ = β_2_ = 0, then the model predicts less or no suppression of $V_{0}^{\infty }$ compared to the “2inactive” condition, since current no longer leaves the hub (arrow indicates net direction of current flow). (D) Hub steady-state membrane potential, $V_{0}^{\infty }$, for varying α_1_, α_2_ and β_1_ values for an inactive spoke 2 (continuous lines; derived from Equation 7 in Supplemental Experimental Procedures) or an ablated spoke 2 (dashed lines; derived from Equation 10 in Supplemental Experimental Procedures). As the plot illustrates, $V_{0}^{\infty }$ is expected to increase with larger α_1_ or smaller α_2_ (fainter lines) and is always smaller when spoke 2 is present compared to when it is ablated. (E) Spoke 1 steady-state membrane potential, $V_{1}^{\infty }$, for varying α_1_, α_2_, and β_1_ values for an inactive spoke 2 (continuous lines; derived from Equation 8 in Supplemental Experimental Procedures) or ablated spoke 2 (dashed lines; derived from Equation 11 in Supplemental Experimental Procedures). Spoke 1 membrane potential is expected to decrease with larger α_1_ and α_2_ (darker lines) and is always smaller when spoke 2 is present compared to when it is ablated.

We formulated a model of a simplified hub-and-spoke circuit (Figure 1A; see also Supplemental Experimental Procedures available online) consisting of a hub interneuron connected to two spoke sensory neurons through electrical synapses (Figure 1A). Since the time course of sensory inputs is substantially slower than the neurons’ electrical time constants, and since *C. elegans* neurons are characterized by graded potentials rather than action potentials, we focused on the steady state rather than the dynamics of the circuit, reasoning that we could derive analytical expressions for the membrane potentials in each neuron of the model circuit. Based on previous findings [9], we assumed the gap junctions to be nonrectifying, and we assumed all neurons to be nonspiking and approximately isopotential, consistent with published electrophysiological data [12]. For simplicity, all cells were electrically passive with similar membrane resistance and capacitance. We derived the steady-state membrane potential in the hub interneuron $\left(V_{0}^{\infty }\right)$ and in the two spoke sensory neurons ($V_{1}^{\infty }$ and $V_{2}^{\infty }$) in response to sensory stimulation in terms of five parameters (Figure 1A; Supplemental Experimental Procedures): the relative gap junction strengths of the two spoke connections (α_1_, α_2_ > 0), the sensory transduction strengths in the input neurons (β_1_, β_2_ > 0), and the receptor reversal potential (*E*^*tr*^ > 0).

We first compared the effects of two simultaneous inputs activating the two spokes (Figure 1A, “2active”) with a single input activating only spoke 1 (i.e., $\beta_{2}^{\prime }\equiv 0$; Figure 1B, “2inactive”). As might be expected, the hub-and-spoke steady-state membrane potentials were smaller for a single input compared to two coinciding inputs for all parameter values, $\forall \alpha_{1},\alpha_{2},\beta_{1}>0$ (Supplemental Experimental Procedures). We also examined the responses to a single input with the second spoke removed altogether (Figure 1C, “2ablated”; $\beta_{2}^{\prime }\equiv 0$ and $\alpha_{2}^{\prime }\equiv 0$). We found that for all parameter values $\forall \alpha_{1},\alpha_{2},\beta_{1}>0$, the ablated circuit had higher activity in the hub and spoke 1 than the circuit with one inactive spoke (Figure 1). That is, the model predicts that if gap junctions are nonrectifying, the response of the circuit should be not only enhanced by coincident activation of multiple spokes but also inhibited by an inactive spoke, whereas there should be less or no effect if a spoke is removed from the circuit altogether. This ability of nonrectifying gap junctions to either transmit current into the hub from an active spoke (Figure 1A) or away from the hub into an inactive spoke (Figure 1B) could facilitate coincidence detection.

**Figure 2:**
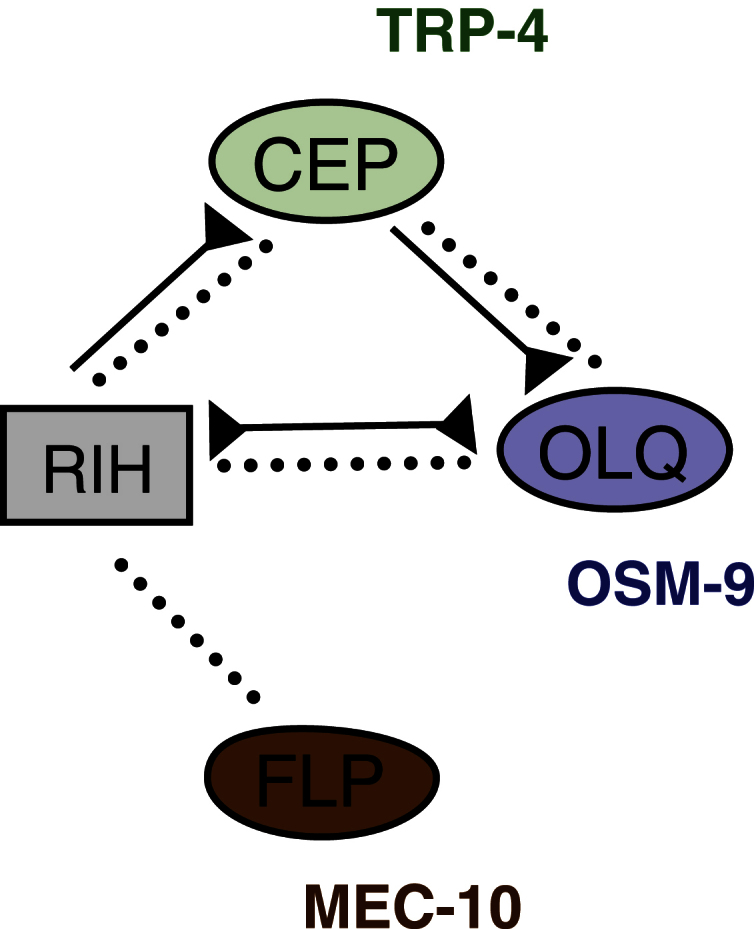
The *C. elegans* Nose Touch Hub-and-Spoke Circuit The nose touch circuit consists of three spoke sensory neuron classes (FLP, OLQ, and CEP) and a hub interneuron (RIH). Distinct proteins required for mechanosensation in each spoke are indicated. Dotted lines represent gap junctions; continuous lines represent chemical synapses.

**Figure 3:**
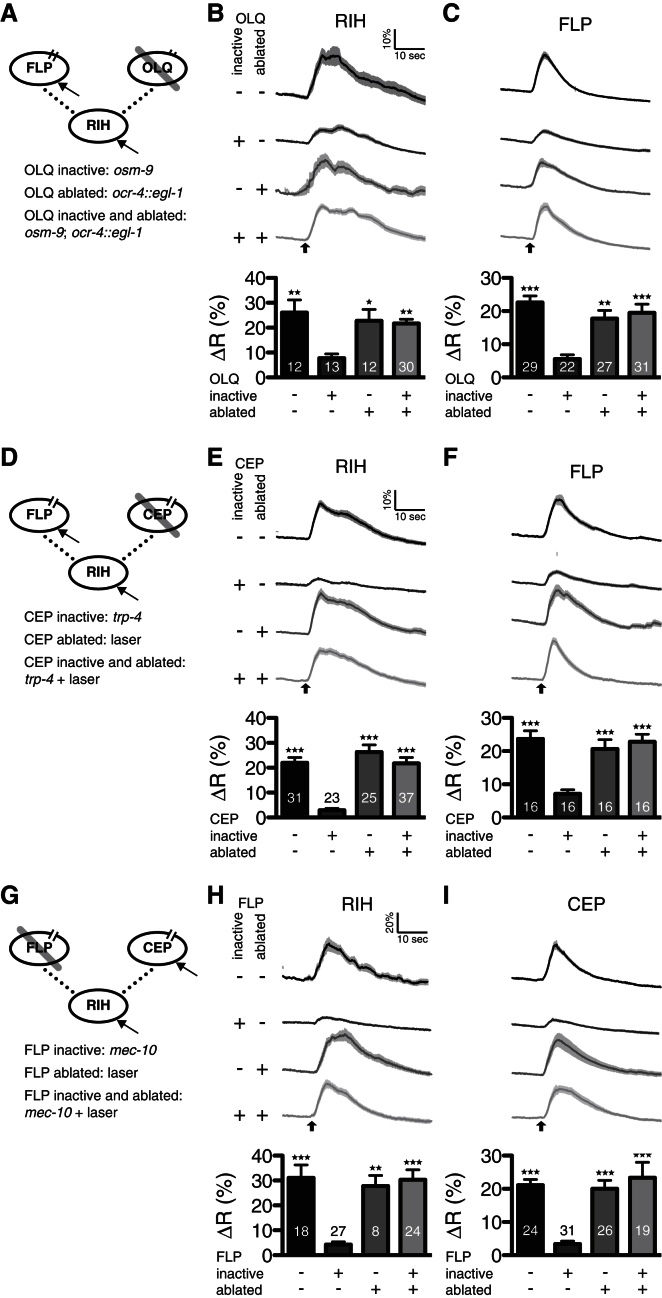
In Vivo Effects of Silencing or Ablating One of the Nose Touch Inputs (A–C) Responses to 2 s nose touch stimuli were recorded in RIH (B; hub) and FLP (C; spoke 1) in wild-type worms and in worms with inactive OLQ (*osm-9* mutant; spoke 2), genetically ablated OLQ (*ocr-4::egl-1*), or both inactive and ablated OLQ. Responses diminished significantly only for inactive but present OLQ. (D–F) Nose touch responses recorded in RIH (E; hub) and FLP (F; spoke 1) in wild-type worms and in worms with inactive CEP (*trp-4* mutant; spoke 2), laser-ablated CEP, or both inactive and ablated CEP. Responses diminished significantly only for inactive but present CEP. (G–I) Nose touch responses recorded in RIH (H; hub) and CEP (I; spoke 1) in wild-type worms and in worms with inactive FLP (*mec-10* mutant; spoke 2), laser-ablated FLP, or both inactive and ablated FLP. Responses diminished significantly only for inactive but present FLP. Numbers in each bar represent the sample size. Error bars represent SEM. ΔR is computed as the percent of the average ratio change 10 s after stimulus onset compared to 10 s just prior to the stimulus onset. Averaged traces include SEM as shaded gray backgrounds. Upward-pointing arrows at the bottom of traces indicate stimulus onset time. ^*^p < 0.05, ^**^p < 0.01, ^***^p < 0.001 relative to the inactive condition by two-tailed unpaired Bonferroni t test.

To test this prediction, we examined the effect of an inactive input on the *C. elegans* nose touch circuit by either silencing a spoke neuron class or ablating it (Figures 2 and 3). In this circuit, three classes of nose touch mechanosensory neurons—two FLPs, four OLQs, and four CEPs—make gap junctions with a single hub, the RIH interneuron (Figure 2). We showed previously [9] that active mechanoreceptors facilitate the responses of other sensory neurons in the network to low-threshold stimuli through gap-junction-mediated lateral facilitation. Nose touch stimulation evokes transient calcium increases in all the sensory neurons, as well as a more sustained calcium transient in RIH. Distinct gene products are required cell autonomously in each mechanoreceptor neuron class for sensing touch (Figure 2): the DEG/ENaC channel MEC-10 in the FLPs [9, 13], the TRPV channel OSM-9 in the OLQs [9, 14], and the TRPN channel TRP-4 in the CEPs [15–17]. We thus imaged nose touch responses in animals with either sensory transduction mutations (*mec-10*, *osm-9*, or *trp-4*) that inactivate or ablations that eliminate the same spoke neurons (Figure 3).

We first compared the effects of inactivating or ablating OLQ (spoke 2) on nose touch responses in RIH (hub) and FLP (active spoke 1; Figures 3A–3C). Consistent with previous results, we observed that mutations in *osm-9* reduced nose touch responses in both RIH and FLP. In contrast, genetically eliminating the OLQs using an *ocr-4::egl-1* transgene did not significantly affect nose touch responses in either RIH or FLP; indeed, eliminating OLQ in an *osm-9* mutant background suppressed the effect of *osm-9* on calcium responses in both RIH and FLP. Similar experiments comparing the effects of inactivating and ablating other spoke neurons yielded similar results. For example, inactivating the CEP neurons through a mutation in *trp-4* (Figures 3D–3F) reduced nose touch responses in both RIH and FLP, whereas laser ablation of the CEP neurons did not; moreover, CEP ablation restored normal nose touch responses in FLP and RIH to the *trp-4* mutant. Likewise, nose touch responses in the RIH and CEP neurons were significantly reduced in FLP-nonresponsive *mec-10* mutant animals, whereas FLP ablation did not significantly affect nose-touch-evoked transients in CEP or RIH in a wild-type background and suppressed the reduced response in the *mec-10* mutant background (Figures 3G–3I). Thus, in all cases, making a class of input neurons nonresponsive inhibited the hub and other spokes, whereas eliminating the same neurons did not. Together, these results support the hypothesis that isolated inputs are suppressed by the hub-and-spoke circuit through shunting.

To further explore the effect of shunting on the response to isolated inputs, we examined the effects of modifying the strength of the electrical connections in the circuit. We modeled the effect of enhancing the relative gap junction coupling strength between an inactive spoke and the hub by multiplying α_2_ by a factor of *m* > 1 in the “2inactive” model (Figure 1B). We found that a larger α_2_ entailed stronger shunting, and therefore lower membrane potential, in both the hub and spoke 1 for all parameter values $\alpha_{1},\alpha_{2},\beta_{1}>0,m>1$, due to an increased current flow into the inactive spoke 2 (Figures 1D and 1E). In contrast, increasing the coupling between an active spoke and the hub (α_1_) reduced spoke membrane potential, since more current could leave this spoke, but resulted in elevated membrane potential at the hub due to an increased current flow from the active spoke 1. We found these predictions to be true for all model parameter values, $\alpha_{1},\alpha_{2},\beta_{1}>0,m>1$.

To test these predictions in the nose touch circuit, we used a transgenic method for experimentally enhancing the gap junction coupling strength between RIH and a subset of its inputs. This approach takes advantage of the fact that connexins, the principal components of vertebrate gap junctions, are not found in invertebrates and are presumably incompatible for the formation of gap junctions with native innexin hemichannels. Thus, ectopic expression of connexin 36 (Cx36) in a subset of *C. elegans* neurons would be expected to introduce new gap junctions between connexin-expressing cells with physically adjacent processes, an expectation supported by tests in other *C. elegans* neurons (unpublished data). Since *cat-1* promoter drives expression in RIH and the CEPs, but not the OLQs or FLPs (Figure S1), a *cat-1::Cx36* transgene would therefore be expected to increase coupling between RIH and the CEPs and potentially shunt current away from the OLQs and FLPs.

**Figure 4:**
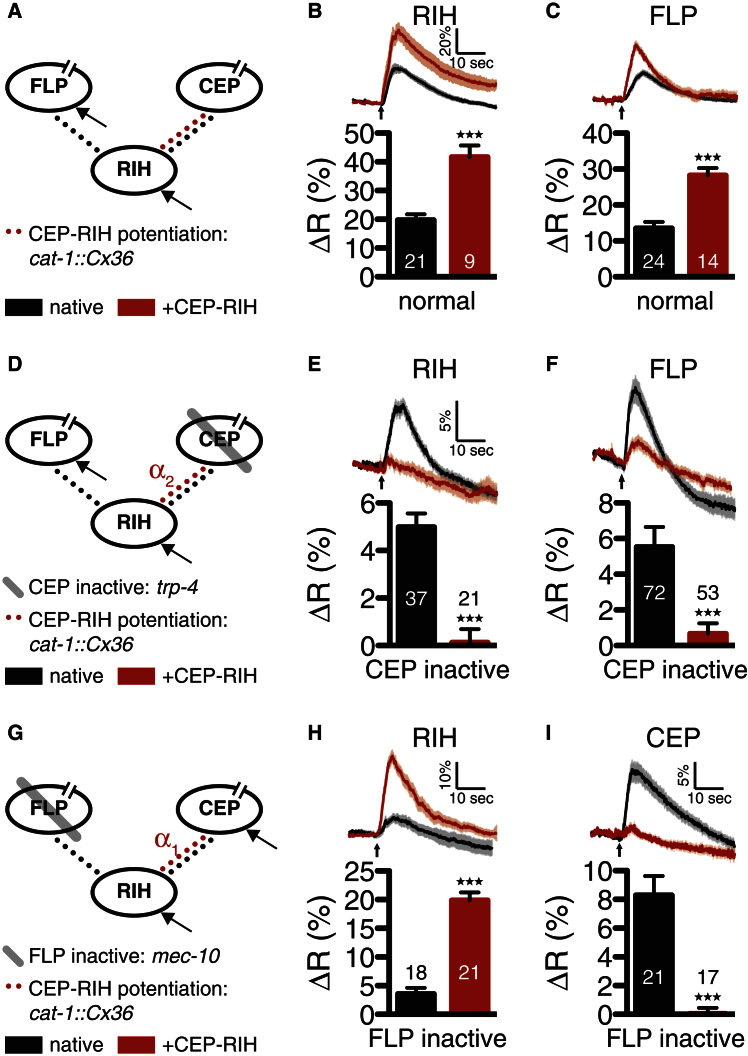
Artificial Strengthening of Spoke-to-Hub Electrical Connections (A–C) The heterologous expression of gap junction proteins in CEP and RIH, expected to strengthen electrical coupling α_1_ between these neurons (A; +CEP-RIH), enhanced the response to a 2 s nose touch stimulus in both RIH (B) and the second spoke, FLP (C), relative to the native circuit. (D–F) When the CEP neuron is inactivated, this heterologous expression (D; +CEP-RIH) further inhibited RIH (E) and the active spoke, FLP (F), as predicted by the model (Figures 2D and 2E). (G–I) When the FLP neuron is inactivated, heterologous expression predicted to increase coupling to the active input CEP (G; +CEP-RIH) increased hub, RIH, activity (H) but inhibited the active spoke, CEP (I), as predicted by the model (Figures 2D and 2E). Numbers in each bar represent the sample size. Error bars represent SEM. ΔR is computed as the mean percent ratio change 10 s after stimulus onset compared to 10 s just prior to the stimulus onset. Averaged traces include SEM as shaded backgrounds. Upward-pointing arrows at bottom of traces indicate stimulus onset time. ^***^p < 0.001 by two-tailed unpaired t test. See also Figures S1 and S2.

To test this possibility, we imaged nose touch responses in RIH and FLP neurons in wild-type and mutant animals carrying the *cat-1::Cx36* array. We observed that in a wild-type background, in which all nose touch inputs are functional, the presence of the *cat-1::Cx36* transgene (Figure 4A) led to significantly larger nose touch responses in the RIH neurons (Figure 4B) as well as in FLP (Figure 4C), as expected if there is increased flow of current into the network from the active CEPs. In contrast, in a *trp-4* mutant background, where the CEP neurons are nonresponsive to touch (Figure 4D), the *cat-1::Cx36* transgene increased the attenuation of nose touch responses in the RIH (Figure 4E) and FLP (Figure 4F) neurons, consistent with increased shunting of current into the inactive CEPs (see also Supplemental Experimental Procedures). Finally, in *mec-10* worms in which FLPs, but not CEPs, are inactive, expression of the *cat-1::Cx36* transgene (Figure 4G) resulted in a diminished CEP response (Figure 4I) but an augmented RIH response (Figure 4H), as predicted by the model when the coupling to the active input is enhanced. Since Cx36 was expressed in monoamine-secreting neurons, we repeated the experiments in *cat-1* mutant worms (defective in the vesicular monoamine transporter) and confirmed that the effects we found were not due to a Cx36-monoamine interaction (Figure S2). Thus, despite some uncertainty about the specific consequences of Cx36 expression in the nose touch circuit, these results provide additional evidence supporting a role for inhibitory shunting in coincidence detection.

## Discussion

We showed previously that sensory neuron activity in the nose touch circuit could enhance the responsiveness of other neurons in the circuit through lateral facilitation [9]. Here we demonstrate that the functionality of the *C. elegans* nose touch circuit relies on the ability of electrical synapses to mediate inhibitory as well as excitatory interactions between neurons. Modeling of a simplified hub-and-spoke circuit suggests that inhibitory shunting by inactive inputs, combined with lateral facilitation, leads to nonlinear amplification of coincident inputs, a prediction supported by cell ablation and neuroimaging experiments in the nose touch circuit. Since coincidence detection emerges from the basic architecture of the hub-and-spoke motif, it seems likely that this microcircuit could perform a similar function in other contexts and in other organisms. Although these studies establish a key role for the hub-and-spoke motif in the nose touch circuit, the real circuit is considerably more complex than the model network. For example, the OLQ and CEP spoke neurons are themselves linked by gap junctions as well as chemical synapses, and the RIH hub neuron also makes feedback chemical synapses with the CEPs (Figure 2). Moreover, both the CEPs and RIH release monoamines that can extrasynaptically modulate other neurons, including mechanoreceptors also involved in nose touch [18, 19]. The integration of these electrical, synaptic, and neuromodulatory networks provides additional complexity to the properties of this small circuit.

What kind of correlations might the nose touch circuit detect? The sensory cilia of the OLQ and CEP neurons are both arranged with fourfold symmetry about the nose, with endings less than 1 μm apart; thus, their spatial receptive fields are most likely very similar. However, the CEP neurons are more deeply embedded in the cuticle than the OLQs, and they use a different sensory transduction mechanism. Therefore, the OLQ and CEP neurons may differ in sensitivity or adaptation properties, and the hub-and-spoke circuit might detect stimulus patterns corresponding to the intersection of the OLQ and CEP tuning curves. In this way, the animals could distinguish between stimuli, such as the texture of a bacterial lawn, that may activate only the CEP neurons [20] and a potentially threatening stimulus coactivating all classes of neurons. Thus, a single circuit could generate distinct behavioral outputs depending on which combination of sensory neurons is active.

These findings provide an explanation for seemingly paradoxical differences between the phenotypes of nervous system mutants and neuronal ablations. For example, mutations in *trpa-1* cause an OLQ-specific defect in nose touch avoidance, whereas OLQ ablation has little or no effect on this behavior [21]. Likewise, *trp-4* loss of function in the CEP neurons causes a defect in nose touch behavior [16], whereas CEP ablation does not [22]. Our results here show that inactive OLQ or CEP neurons inhibit nose touch responses in the RIH and FLP neurons whereas ablations of these neurons have little effect; thus, inhibition caused by shunting to inactive neurons appears to explain the *trpa-1* and *trp-4* nose touch phenotypes. Similar results have been observed in other circuits; for example, loss of *trp-4* in the proprioceptive DVA neurons has been reported to cause aberrant locomotion behavior, whereas ablation of the DVA neurons has no locomotion defect and suppresses the *trp-4* mutant phenotype [15]. Since DVA is linked to both locomotory interneurons and motor neurons, we hypothesize that in *trp-4* mutants, inactive DVA neurons suppress activity in the locomotion circuit through shunting. These results suggest that, in at least some cases, neuronal activation or silencing experiments may provide a more sensitive method for identifying neurons involved in a particular circuit or behavior than cell ablation experiments.

In a sense, the hub-and-spoke architecture can be viewed as analogous to the structure of neocortical neurons, with the hub corresponding to the soma, the spokes to dendritic branches with synaptic inputs [23], and the gap junctions to the axial resistance along the dendrites. In neurons, dendritic branching supports compartmentalized processing of synaptic signaling [24] and has particularly been shown to underlie coincidence detection, for example in auditory brainstem neurons [25]. By analogy, and due to very similar biophysical principles, the distributed sensory receptors on the spokes seem to instantiate a compartmentalized sensory module, enhancing the sensitivity of the circuit to a broad range of stimulus intensities and enabling coincidence detection.

## Experimental Procedures

### C. *elegans* Strains

A detailed strain list is provided in the Supplemental Experimental Procedures.

### Theoretical Model of Hub-and-Spoke Circuit

Details of the theoretical model for the hub-and-spoke circuit appear in the Supplemental Experimental Procedures.

### Calcium Imaging

Calcium imaging of nose touch stimulation was performed essentially as described previously [21, 26]. For details, see the Supplemental Experimental Procedures.

## Supplemental Information

Document S1. Figures S1 and S2 and Supplemental Experimental Procedures
