# Supplementary material for: A Gap Junction Circuit Enhances Processing of Coincident Mechanosensory Inputs
Source: Curr Biol. 2013 Jun 3;23(11):963–7. doi: 10.1016/j.cub.2013.04.030 (PMC3675673; doi:10.1016/j.cub.2013.04.030)
Supplement: Document S1. Figures S1 and S2 and Supplemental Experimental Procedures [file mmc1.pdf]

Current Biology, Volume *23*

Supplemental Information

A Gap Junction Circuit Enhances Processing of Coincident Mechanosensory Inputs

Ithai Rabinowitch, Marios Chatzigeorgiou, and William R. Schafer

Supplemental Inventory

Figure S1. Expression of Cx36 in the nose touch circuit, related to Figure 4

Figure S2. Ectopic gap junction connections created by expressing Cx36 in the nose touch circuit, related to Figure 4

Supplemental Experimental Procedures

Supplemental References

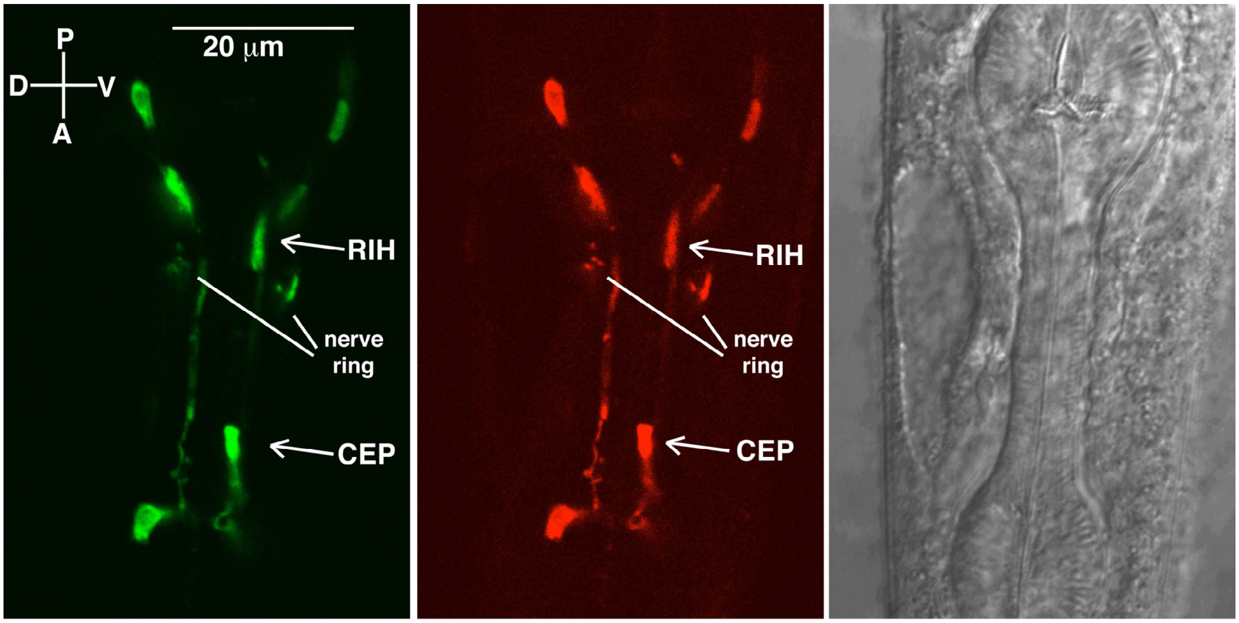

Figure S1. Expression of Cx36 in the Nose Touch Circuit

Shown are fluorescence (left and middle panels) and DIC (right panel) images of AQ2792 (*ljIs103[cat-1::YCD3]; ljEx400[cat-1::Cx36*::mCherry, unc-122::mCherry]*) animals, showing expression of yellow cameleon YCD3 (left panel) and mCherry-tagged Cx36 (middle panel) in the CEP and RIH neurons (labeled with arrows). Nerve ring fluorescence is just below (i.e. anterior) to the RIH cell body in the middle panel. Images were taken with a Zeiss 780 microscope, with a Plan Apochromat 63x/1.4 oil lens, DIC filters BP 505-530 and LP560, beam splitters 488/543, NFT 545.

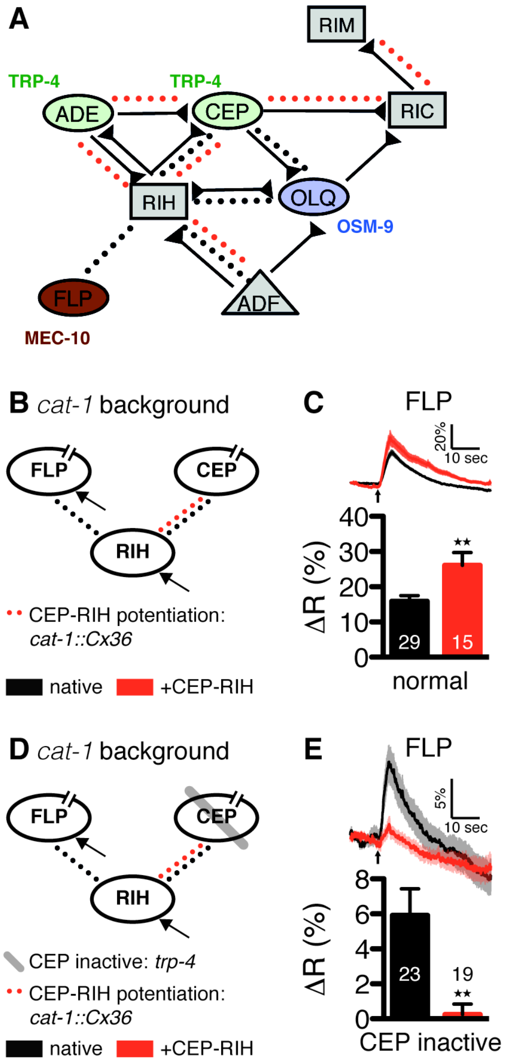

Figure S2. Ectopic Gap Junction Connections Created by Expressing Cx36 in the Nose Touch Circuit

(A) Expected expression pattern of Cx36 under the *cat-1* promoter. Black lines with triangles at ends represent native chemical synapses. Black dotted lines represent native electrical synapses. Red dotted lines indicate putative newly formed Cx36 electrical synapses. Although we expect several new gap junctions to be added between various neurons, the targeted connection is between CEP and RIH.

(B and C) The insertion of an additional gap junction between the spoke, CEP, and the hub, RIH, in worms defective in serotonin and dopamine neurotransmission (*cat-1* mutants) enhanced the response to nose touch in the second spoke, FLP (C), in a similar manner to wild type worms (Figure 4C).

(D and E) Strengthening the electrical connection between the inactive spoke, CEP, and the hub, RIH, in *cat-1* mutant worms increased the extent of inhibition of the active spoke, FLP (E), similar to wild type (Figure 4F). Arrows below traces in C and E indicate stimulation onset.

Numbers in each bar represent the sample size. Averaged traces include SEM as shaded background. Error bars represent SEM. $^{**}p < 0.01$ by two-tailed unpaired t-test.

Supplemental Experimental Procedures

Strain List

AQ2044 *ljIs103[cat-1::YCD3].*
AQ2860 *osm-9(ky10);ljIs103[cat-1::YCD3] line1.*
AQ2732 *ljIs103[cat-1::YCD3];ljEx403[ocr-4::egl-1;elt-2::rfp].*
AQ2859 *osm-9(ky10);ljIs103[cat-1::YCD3];ljEx403[ocr-4::egl-1;elt-2::rfp].*
AQ2145 *ljEx217[egl-46::YC2.3].*
AQ2148 *osm-9(ky10); ljEx217[egl-46::YC2.3].*
AQ2733 *ljEx217[egl-46::YC2.3];ljEx403[ocr-4::egl-1;elt-2::rfp].*
AQ2915 *osm-9(ky10); ljEx217[egl-46::YC2.3];ljEx403[ocr-4::egl-1(d);elt-2::rfp].*
AQ2815 *trp-4(gk341); ljIs103[cat-1::YCD3].*
AQ2814 *trp-4(gk341); ljEx217[egl-46::YC2.3].*
AQ2697 *mec-10(tm1552);ljIS104[cat-1::YCD3].*
AQ2792 *ljIs103[cat-1::YCD3]; ljEx400[cat-1::Cx36*::mCherry, unc-122::mCherry].*
AQ2639 *ljEx217[egl-46::YC2.3]; ljEx400[cat-1::Cx36*::mCherry, unc-122::mCherry].*
AQ2812 *trp-4(gk341); ljIs103[cat-1::YCD3]; ljEx400[cat-1::Cx36*::mCherry, unc-122::mCherry].*
AQ2813 *trp-4(gk341); ljEx217[egl-46::YC2.3]; ljEx400[cat-1::Cx36*::mCherry, unc-122::mCherry].*
AQ2914 *mec-10(tm1552);ljIS104[cat-1::YCD3]; ljEx400[cat-1::Cx36*::mCherry, punc-122::mCherry].*
AQ2924 *cat-1(e1111); ljEx217[egl-46::YC2.3].*
AQ2925 *cat-1(e1111); ljEx217[egl-46::YC2.3]; ljEx400[cat-1::Cx36*::mCherry, punc-122::mCherry].*
AQ2926 *cat-1(e1111); trp-4(gk341); ljEx217[egl-46::YC2.3].*
AQ2927 *cat-1(e1111); trp-4(gk341); ljEx217[egl-46::YC2.3]; ljEx400[cat-1::Cx36*::mCherry, punc-122::mCherry].*

Calcium Imaging

Calcium imaging of nose touch stimulation was performed by probing the worm's nose under the imaging microscope with a motor-controlled glass capillary for 2 s. Images were recorded at 10 Hz using an iXon EM camera (Andor Technology) and captured using IQ1.9 software (Andor Technology). Analysis was done using a custom written Matlab (MathWorks) program. A rectangular region of interest (ROI) was drawn surrounding the cell body and for every frame the ROI was shifted according to the new position of the centre of mass. Fluorescence intensity, F, was computed as the difference between the sum of pixel intensities and the faintest 10% pixels (background) within the ROI. Fluorescence ratio $R=F_Y/F_C$ of the yellow and cyan channels after correcting for bleed through was used for computing ratio change, ΔR. ΔR for calcium traces was equal to $(R-R_0)/ R_0*100$, where R_0 is the average R within the first 3 sec of recording. For statistical quantification ΔR was computed as $(R_1-R_0)/ R_0*100$, where R_0 and R_1 are the average R over 10 sec prior and following nose touch stimulation. A two-tailed unpaired t-test was used to analyze the data. Where more than one comparison was made, an ANOVA followed by Bonferroni t-tests were used instead.

Ectopic Cx36 Expression

The cDNA sequence of *Mus musculus* gap junction protein, delta 2 (Gjd2) was codon optimized to produce a synthetic *Cx36* gene (GeneArt). We fused this *Cx36** gene to an upstream *cat-1* promoter and a downstream gene encoding mCherry. The plasmid was injected at 70 ng/µl together with a coinjection marker, *unc-122::mCherry*, to generate transgenic worms carrying the *cat-1::Cx36** gene on an extrachromosomal array (*ljEx400*).

Model of Two Spoke Neurons Connected to a Hub Neuron

In order to investigate the function of hub and spoke circuits we formulated a simplified current-based model (Figure 1A). Many previous studies have used similar models mainly to study the impact of gap junctions on network oscillations and synchrony (e.g. [1–5]). Whereas most previous modeling work focused on the dynamics of the network, we were interested in the steady state voltage response of participating neurons. The reason was that our model was designed to compare the effects of combined versus isolated sensory inputs, with a time course that is substantially slower than the neurons' time constants. Thus, we concentrated on the coincidence of sensory stimuli rather than the coincidence of action potentials. Furthermore, the activity of *C. elegans* neurons is characterized mostly by graded potentials rather than by spiking temporal patterns, which allowed us to ignore discreet brief electrical events at faster timescales. For simplicity we considered the neurons in the circuit to be passive and isopotential with equal membrane conductance (G_m), capacitance (C_m) and time constant ($\tau = C_m/G_m$). The model considers the gap junctions to be non-rectifying bi-directional and does not account for additional synapses within the circuit or for connections with other non-circuit neurons. The following system of equations describes V_i, the membrane potential minus resting potential of the hub (i=0) and each spoke (i=1,2):

$$\tau \frac{dV_0}{dt} = -V_0 - (V_0 - V_1)\alpha_1 - (V_0 - V_2)\alpha_2 \qquad (1)$$

$$\tau \frac{dV_1}{dt} = -V_1 - (V_1 - V_0)\alpha_1 - (V_1 - E^{tr})\beta_1 \qquad (2)$$

$$\tau \frac{dV_2}{dt} = -V_2 - (V_2 - V_0)\alpha_2 - (V_2 - E^{tr})\beta_2 \qquad (3)$$

In these equations α_i represent the relative gap junction coupling strengths between the hub and each spoke i, defined as the ratio between the gap junction conductance, $g_i^{gj} > 0$, and the membrane conductance, $\alpha_i \equiv g_i^{gj}/G_m > 0$. β_i represent the relative receptor transduction strength of each sensory neuron i, defined as the ratio between the receptor conductance $g_i^{tr} > 0$, assumed to be proportional to stimulus intensity and to be constant in time, and membrane conductance, $G_m > 0$, $\beta_i \equiv g_i^{tr}/G_m > 0$. E^{tr} is the sensory receptor reversal potential minus resting potential. The steady state solutions to Equations 1-3 are given by:

$$V_0^\infty = \frac{\alpha_1\beta_1(1+\alpha_2)+\alpha_2\beta_2(1+\alpha_1)+\beta_1\beta_2(\alpha_1+\alpha_2)}{1+2\alpha_1+2\alpha_2+3\alpha_1\alpha_2+\beta_1(1+\alpha_1+2\alpha_2+\alpha_1\alpha_2)+\beta_2(1+2\alpha_1+\alpha_2+\alpha_1\alpha_2)+\beta_1\beta_2(1+\alpha_1+\alpha_2)} \cdot E^{tr} \qquad (4)$$

$$V_1^\infty = \frac{\alpha_1 V_0^\infty + \beta_1 E^{tr}}{1+\alpha_1+\beta_1} \qquad (5)$$

$$V_2^\infty = \frac{\alpha_2 V_0^\infty + \beta_2 E^{tr}}{1+\alpha_2+\beta_2} \qquad (6)$$

Noncoincident Spoke Activation Results in Shunting

The hub and spoke circuit is wired to receive several different inputs from its various spoke sensory neurons. We wished to examine the circuit's response to a single input. We thus analyzed, without loss of generality, the response of the hub to an input received in spoke 1, while spoke 2 is inactive ('2inactive'; Figure 1B). To implement this condition in the model we set the receptor strength of spoke 2 to $\beta_2^{'} \equiv 0$ and obtained the following steady state solution:

$$V_{0,2inactive}^{\infty} = \frac{\alpha_1\beta_1(1+\alpha_2)}{1+2\alpha_1+2\alpha_2+3\alpha_1\alpha_2+\beta_1(1+\alpha_1+2\alpha_2+\alpha_1\alpha_2)} \cdot E^{tr} \quad (7)$$

$$V_{1,2inactive}^{\infty} = \frac{\alpha_1 V_{0,2inactive}^{\infty}+\beta_1 E^{tr}}{1+\alpha_1+\beta_1} = \frac{(1+\alpha_1+2\alpha_2+\alpha_1\alpha_2)}{\alpha_1(1+\alpha_2)} \cdot V_{0,2inactive}^{\infty} \quad (8)$$

As might be expected, the steady state membrane potential at the hub was smaller for a single input compared to two coinciding inputs. This was true for all parameter values, $\forall \alpha_1, \alpha_2, \beta_1 > 0$:

$$\frac{V_{0,2inactive}^{\infty}-V_0^{\infty}}{V_0^{\infty}} = \frac{-\alpha_2(1+\alpha_1+\beta_1)((1+2\alpha_2)(1+\beta_1)+\alpha_1(2+3\alpha_2+\beta_1))\beta_2}{((1+2\alpha_2)(1+\beta_1)+\alpha_1(2+\beta_1+\alpha_2(3+\beta_1)))(\alpha_2(1+\beta_1)\beta_2+\alpha_1(\alpha_2\beta_2+\beta_1(1+\alpha_2+\beta_2)))} < 0 \quad (9)$$

The obvious reason for this diminished response could be the mere reduction in the number of inputs arriving at the hub. In order to determine whether this was the only cause for the decrease in the hub response, or whether it was also due to the properties of the circuit, we examined a second condition, whereby the inactive spoke 2 was removed altogether from the circuit ('2ablated'; Figure 1C). The solutions for an ablated spoke 2, attained by setting $\beta_2^{'} \equiv 0$ but also $\alpha_2^{'} \equiv 0$ are:

$$V_{0,2ablated}^{\infty} = \frac{\alpha_1\beta_1}{1+2\alpha_1+(1+\alpha_1)\beta_1} \cdot E^{tr} \quad (10)$$

$$V_{1,2ablated}^{\infty} = \frac{\alpha_1 V_{0,2ablated}^{\infty}+\beta_1 E^{tr}}{1+\alpha_1+\beta_1} = \frac{1+\alpha_1}{\alpha_1} \cdot V_{0,2ablated}^{\infty} \quad (11)$$

Comparing between the two scenarios, we found that for all parameter values $\forall \alpha_1, \alpha_2, \beta_1 > 0$, inactivating spoke 2 and thus de-correlating the two spokes, has a larger inhibitory effect on the hub and on spoke 1, than does just ablating spoke 2:

$$\frac{V_{0,2inactive}^{\infty}-V_{0,2ablated}^{\infty}}{V_{0,2ablated}^{\infty}} = \frac{-\alpha_2(1+\alpha_1+\beta_1)}{(1+2\alpha_2)(1+\beta_1)+\alpha_1(2+\beta_1+\alpha_2(3+\beta_1))} < 0 \quad (12)$$

$$\frac{V_{1,2inactive}^{\infty}-V_{1,2ablated}^{\infty}}{V_{1,2ablated}^{\infty}} = \frac{-\alpha_1^2\alpha_2}{(1+\alpha_1)((1+2\alpha_2)(1+\beta_1)+\alpha_1(2+\beta_1+\alpha_2(3+\beta_1)))} < 0 \quad (13)$$

Thus, the diminished response to a single uncorrelated input is due, in addition to the reduction in the number of inputs, also to a unique feature of the gap junction circuit. We hypothesized that the mechanism underlying this suppression of non-coincident inputs is based on shunting, whereby current is drawn away from the hub, and indirectly also from the active spoke 1, to the inactive spoke 2. Such shunting of single non-coinciding inputs is an important component necessary for coincidence detection.

Individual Contribution of Each Gap Junction in the Circuit to Shunting

To further establish whether the diminished single input response is due to shunting, and to appreciate the individual contribution of each gap junction in the circuit to such shunting we plotted V_0^∞ / E^{Tr} (Figure 1D; main text) and V_1^∞ / E^{Tr} (Figure 1E; main text) as a function of α_1 and representative values of α_2 and β_1 for the '2inacitve' and '2ablated' conditions. In both neurons the degree of inhibition increased (V_0^∞ / E^{Tr} and V_1^∞ / E^{Tr} became smaller) with greater α_2 values (Fig. 1D,E), suggesting a larger current flow into spoke 2 and thus more shunting. Greater α_1 values also resulted in a decrease in the active spoke 1 membrane potential (Fig. 1E), since more current could leave it towards spoke 2. However, the hub membrane potential actually increased for larger α_1 values (Fig. 1D), since spoke 1 became more effective in depolarizing the hub. These observations are consistent with the hypothesis that an inactive spoke associated with an uncorrelated input, may introduce shunting to the circuit.

In order to systematically examine these findings in the '2inactive' model we studied how modifying the strength of each circuit gap junction may affect steady state membrane potential in the neurons. First, we considered the effects of enhancing (‘*’) the relative gap junction coupling strength between inactive spoke 2 and the hub, by multiplying α_2 by a factor of $m>1$,

$$V_{0,2inactive*}^\infty = \frac{\alpha_1\beta_1(1+m\alpha_2)}{1+2\alpha_1+2m\alpha_2+3\alpha_1 m\alpha_2+\beta_1(1+\alpha_1+2m\alpha_2+\alpha_1 m\alpha_2)}\cdot E^{tr} \quad (14)$$

$$V_{1,2inactive*}^\infty = \frac{(1+\alpha_1+2m\alpha_2+\alpha_1 m\alpha_2)}{\alpha_1(1+m\alpha_2)}\cdot V_{0,2inactive*}^\infty \quad (15)$$

Indeed, a larger α_2 entailed stronger shunting of both the hub and spoke 1 for all parameter values $\alpha_1,\alpha_2,\beta_1>0,\ m>1$:

$$\frac{V_{0,2inactive*}^\infty - V_{0,2inactive}^\infty}{V_{0,2inactive}^\infty} = \frac{-(m-1)\alpha_2(1+\alpha_1+\beta_1)}{(1+\alpha_2)((1+\beta_1)+(1+2m\alpha_2)+\alpha_1(2+\beta_1+m\alpha_2(3+\beta_1))}<0 \quad (16)$$

$$\frac{V_{1,2inactive*}^\infty - V_{1,2inactive}^\infty}{V_{1,2inactive}^\infty} = \frac{-(m-1)\alpha_1^2\alpha_2}{(1+\alpha_1+\alpha_2(2+\alpha_1))((1+\beta_1)(1+2m\alpha_2)+\alpha_1(2+\beta_1+m\alpha_2(3+\beta_1)))}<0 \quad (17)$$

However, strengthening the relative coupling, α_2, between the active spoke 1 and the hub,

$$V_{0,2inactive,1*}^\infty = \frac{m\alpha_1\beta_1(1+\alpha_2)}{1+2m\alpha_1+2\alpha_2+3m\alpha_1\alpha_2+\beta_1(1+m\alpha_1+2\alpha_2+m\alpha_1\alpha_2)}\cdot E^{tr} \quad (18)$$

$$V_{1,2inactive,1*}^\infty = \frac{(1+m\alpha_1+2\alpha_2+m\alpha_1\alpha_2)}{m\alpha_1(1+\alpha_2)}\cdot V_{0,2inactive,1*}^\infty \quad (19)$$

increased the shunting of spoke 1, but reduced the shunting of the hub:

$$\frac{V_{0,2inactive,1*}^\infty - V_{0,2inactive}^\infty}{V_{0,2inactive}^\infty} = \frac{(m-1)(1+2\alpha_2)(1+\beta_1)}{1+\beta_1+2m\alpha_1+m\alpha_1\beta_1+\alpha_2(2+3m\alpha_1+\beta_1(2+m\alpha_1))}>0 \quad (20)$$

$$\frac{V_{1,2inactive,1*}^\infty - V_{1,2inactive}^\infty}{V_{1,2inactive}^\infty} = \frac{-(m-1)\alpha_1(1+2\alpha_2)^2}{(1+\alpha_1+2\alpha_2+\alpha_1\alpha_2)(1+\beta_1+2m\alpha_1+m\alpha_1\beta_1+\alpha_2(2+3m\alpha_1+\beta_1(2+m\alpha_1)))}<0 \quad (21)$$

These results support the hypothesis that a selective shunting mechanism is intrinsic to hub and spoke circuits, and may sharpen the distinction between single uncoordinated inputs and overall coinciding ones.

Supplemental References

1. Kepler, T. B., Marder, E., and Abbott, L. F. (1990). The effect of electrical coupling on the frequency of model neuronal oscillators. Science *248*, 83–85.
2. Kistler, W. M., and De Zeeuw, C. I. (2005). Gap junctions synchronize synaptic input rather than spike output of olivary neurons. In Progress in Brain Research, De Zeeuw & Cicirata, ed. (Elsevier), pp. 189–197. Available at: http://www.sciencedirect.com/science/article/pii/S0079612304480169 [Accessed March 20, 2013].
3. Bem, T., Meyrand, P., Branchereau, P., and Hallam, J. (2008). Multi-Stability and Pattern-Selection in Oscillatory Networks with Fast Inhibition and Electrical Synapses. PLoS ONE *3*, e3830.
4. Hjorth, J., Blackwell, K. T., and Kotaleski, J. H. (2009). Gap junctions between striatal fast-spiking interneurons regulate spiking activity and synchronization as a function of cortical activity. J. Neurosci. *29*, 5276–5286.
5. Publio, R., Oliveira, R. F., and Roque, A. C. (2009). A Computational Study on the Role of Gap Junctions and Rod Ih Conductance in the Enhancement of the Dynamic Range of the Retina. PLoS ONE *4*, e6970.
